# A novel Bursin-like peptide as a potential virus inhibitor and immunity regulator in SPF chickens infected with recombinant ALV

**DOI:** 10.1186/s12917-019-2192-2

**Published:** 2019-12-10

**Authors:** Yukun Zeng, Zuxin Gong, Binbin Wu, Wenchao Guan, Shenyi Yu, Yajuan An, Rongbin Lu, Jinrong Zhao, Yijian Wu, Yifan Huang, Xiaoping Wu

**Affiliations:** 1 College of Animal Science, Fujian Agricultural and Forestry University, Fuzhou, 350002 People’s Republic of China; 2 Fujian Key Laboratory of Traditional Chinese Veterinary Medicine and Animal Health, Fujian Agricultural and Forestry University, Fuzhou, 350002 People’s Republic of China; 3 School of Basic Medical Sciences, Fujian Medical University, Fuzhou, 350004 People’s Republic of China

**Keywords:** Bursin-like peptide, ALV, Immunity suppression, Growth, Virus inhibitor

## Abstract

**Background:**

Avian leukosis viruses (ALVs) are important contagious suppressive factors of chicken immunity and growth performance, resulted in enormous economic loss. Although virus eradication programs are applied in breeder flocks, ALVs are still widespread globally. Therefore, other valuable adjunct to reduce the negative effect of ALVs should be considered. Bursin-like peptide (BLP) showed remarkable immunomodulatory effects, whereas their influence on ALV-infected avian groups has not been reported. Here, a designed hybrid BLP was expressed in *E. coli*. The purified BLP was injected subcutaneously weekly in SPF chickens congenitally infected with a natural ALV strain. Then the influences of this BLP on the growth performance, immune response and virus titer of ALV-infected chickens were determined.

**Results:**

This BLP injection significantly improved the body weights of ALV-infected birds (*P* < 0.05). BLP injection significantly enhanced organ index in the BF in ALV-infected birds (*P* < 0.05). The weekly injection of BLP significantly lengthened the maintenance time of antibodies against Newcastle disease virus (NDV) attenuated vaccine of ALV-infected birds (*P* < 0.05) and boosted the antibody titer against avian influenza virus (AIV) H_5_ inactive vaccine of mock chicken (*P* < 0.05). BLP injection in mock chickens enhanced the levels of serum cytokines (IL-2, IL-4 and interferon-γ) (*P* < 0.05). Surprisingly, the novel BLP significantly inhibited expression of the ALV *gp85* gene in the thymus (*P* < 0.05), kidney (*P* < 0.05) and bursa of Fabricius (BF) (*P* < 0.01) of ALV-infected chickens. Both viral RNA copy number and protein level decreased significantly with BLP (50 μg/mL) inoculation before ALV infection in DF1 cells (*P* < 0.05).

**Conclusions:**

This is the first report investigating the influence of BLP on the growth and immunity performance of chickens infected by ALV. It also is the first report about the antiviral effect of BLP in vivo and in vitro. This BLP expressed in *E. coli* showed potential as a vaccine adjuvant, growth regulator and antiretroviral drug in chickens to decrease the negative effects of ALV infection.

## Background

Avian leukosis viruses (ALVs) are divided into 6 subgroups (A, B, C, D, E and J) in chicken flocks. It can cause diverse tumors [1], immunity suppression [2] and growth performance inefficiency [3], resulting in great economic losses. Until now, various eradication and control programs have been successfully developed and applied, including antibody testing, antigen testing, virus isolation [1], hatching and rearing chickens in small groups [4], and different vaccine preparations [5]. Nonetheless, ALV infections still have been reported worldwide [6]. As such, other adjuncts to virus eradication programs in the field should be considered to reduce economic losses caused by ALVs.

Bursin (BS), a tripeptide (Lys-His-Gly-NH_2_) hormone [7], is known to selectively stimulate avian B cell differentiation [8] and promote immune-globulin (Ig) switching from IgM to IgG [9]. A novel stabilized bursin mimetic, Gagnon’s tetrapeptide (Lys-Asn-Pro-Tyr), not only induces the selective expansion of B cells but also stimulates cytotoxic T lymphocytes [10]. A series of synthetic bursin and bursin-like fusion peptides (BLPs) were used as potential vaccine adjuvants in a Japanese encephalitis virus (JEV) subunit vaccine [11] and a H_9_N_2_ avian influenza virus (AIV) inactivated vaccine [12] in mice, as well as in an infectious bursal disease virus (IBDV) inactivated vaccine in chickens [13]. Nonetheless, previous studies have mainly focused on the immunomodulatory effects of BS or BLP on mock animals and there is a lack of representation from actual flocks with various immune suppression factors. As far as is known, the influence of BS or BLPs on avian groups with ALVs infection has not been reported, even though ALVs are provided with important effects on chicken immunity and growth.

Herein, we hypothesized that a novel recombinant hybrid polypeptide consisting of a tandem array of Gagnon’s peptide and albumin-binding peptide, heterologously expressed in *E. coli* would have significant immunological activity. We closely mimicked the route of congenital infection of ALV recombinant strain FJ15HT0 in SPF chicken embryos and then injected this purified BLP weekly in ALV-infected and mock control chickens. The influence of the BLP on the growth and humoral response of chickens infected with ALV was discussed, and then the effect of this BLP on ALV replication in vitro and in vivo was evaluated.

## Results

### Heterologous expression of fusion BLP

**Fig. 1:**
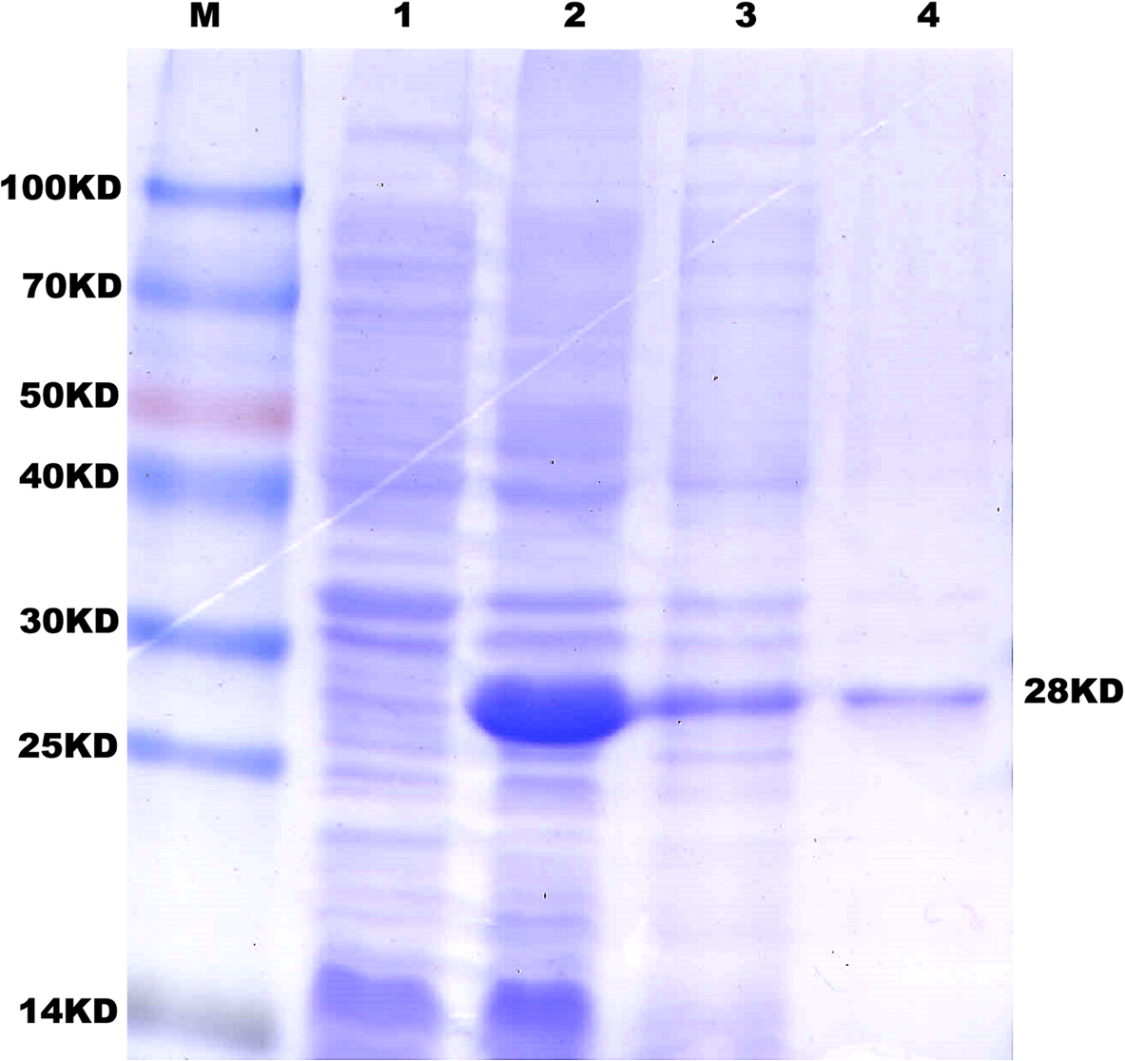
Heterologously expression and purification of BLP*.* Lane M standard protein molecular-weight marker, **Lane 1** the control, **Lane 2** the expression products of recombinant *E. coli* containing the hybrid polypeptide DNA, **Lane 3** the supernate of the recombinant *E. coli*, Lane 4 purified recombinant BLP

Considering the molecular weight of the BLP was 8 kDa and the molecular weight of the fusion tag peptide from pET32a(+) was approximately 20 kDa, the theoretical molecular weight of fusion protein should be approximately 28 kDa. Following induction with IPTG, a protein band between 25 kDa and 30 kDa was seen via SDS-PAGE (Fig. 1), indicating that the fusion protein was successfully expressed in *E. coli* BL21 (DE3). The soluble fusion protein was purified from *E. coli* culture supernatants using Ni^2+^-chelating affinity chromatography. After desalting and concentration, the concentration of the purified BLP was approximately 500 μg/mL, as measured by BCA protein assay kit.

### Effect of BLP on the body weight of ALV infected chickens

**Fig. 2:**
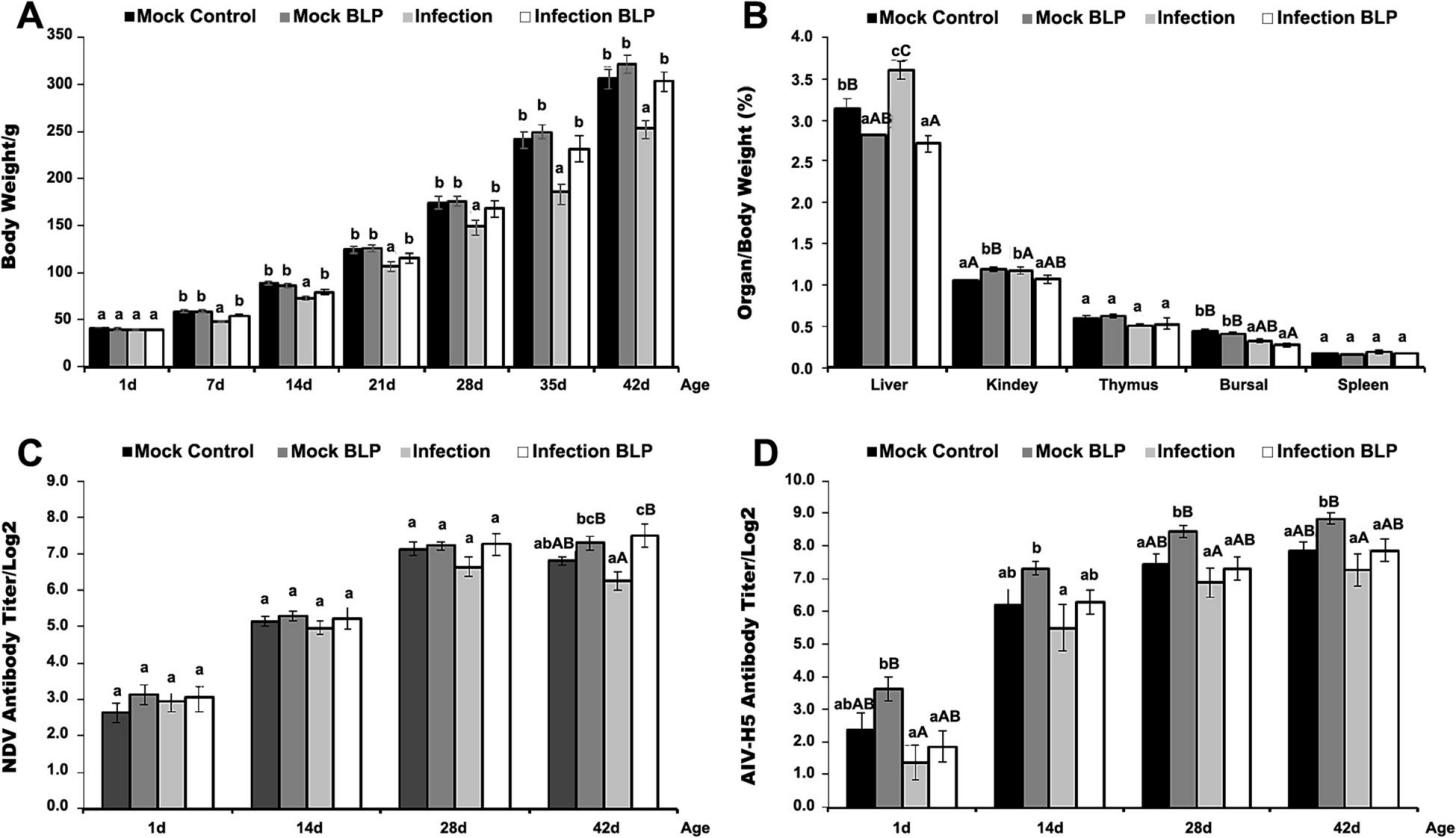
Effect of BLP on the Body Weight and Immunity Performance of ALV-infected Chickens. A Body weights of chickens in different groups (g). **B** Effect of BLP on the Organ Index of Chickens (Mean ± SE, %). **C** Serum antibody titer against NDV active vaccine of the chickens in different groups (Log2). **D** Serum antibody titer against AIV inactive vaccine of the chickens in different groups (Log2). Note: The different lowercases and capitals indicated significant difference at level of *P* < 0.05 and *P* < 0.01

As shown in Fig. 2a, the simulated congenital infection with ALV FJ15HT0 strain alone resulted in significant growth suppression from day 7 to 42 of age, compared with the two mock groups (*P* < 0.05). Injection of BLP in mock SPF chickens showed no significant influence on growth (*P* > 0.05). Interestingly, injection with purified BLP weekly exerted a significant moderating effect on growth performance from day 7 to 42 of age in ALV-infected chickens, compared with those ALV infected controls (*P* < 0.05).

### Effect of BLP on the organ index of chickens

At 42 d of age, samples of liver, kidney, thymus, spleen and BF were excised from all chicks and weighed to establish immune organ indices, as shown in Fig. 2b. This novel BLP injection significantly reduced the organ index of liver both in mock (*P* < 0.05) and ALV-infected chickens (*P* < 0.01), reduced the kidney organ index in infected birds (*P* < 0.01) and decreased organ index in the BF in ALV-infected birds (*P* < 0.05). These results indicated that BLP modulated liver and kidney swelling, as well as BF atrophy induced by ALV infection.

### Effect of BLP on antibody titer against NDV live vaccine and AIV inactivated vaccine

At 1 d and 14 d of age, all experimental birds were simultaneously inoculated with NDV live attenuated vaccine (via eye and nasal drops) and AIV inactivated vaccine (subcutaneously). Serum antibody titers against NDV and AIV were then assessed by HI.

As shown in Fig. 2c, no significant differences were observed for antibody responses to NDV vaccination between the four groups (*P* > 0.05) from days 1 to 28 of age. Nonetheless, BLP injection showed a significant promotion of NDV antibody titer of ALV-infected birds at 42 d of age, compared with the infection control (*P* < 0.01) and the mock control groups (*P* < 0.05), suggesting the BLP showed no effect on the peak antibody titer value against the NDV live vaccine, but significantly affected the maintenance time of its peak titer in ALV infected chickens. As shown in Fig. 2d, although BLP showed no significant effect on the antibody response of ALV-infected hosts to the AIV inactivated vaccine (*P* > 0.05) (1d - 42d), it was obvious that the BLP significantly improved the antibody response of chickens in Mock BLP group to inactivated AIV vaccine, compared with the birds in Infection Control group (*P* < 0.05) (14d - 42d), and also compared with those in Mock Control group (*P* < 0.05) (28d - 42d).

### Effect of BLP on serum cytokine titer

The results indicated that ALV infection alone and BLP inoculation alone in chickens invoked significantly higher serum IL-2, IL-4, and IFN-γ responses (*P* < 0.05 or *P* < 0.01) at 14 d and 28 d compared with mock control chickens, albeit to differing degrees, as shown in Table 1.

**Table 1:** Effect of BLP on the Serum Cytokines of Chickens (Mean ± SE, pg/mL)

Cytokines Treatment	IL2		IL4		IFN-γ	
	35d	42d	35d	42d	35d	42d
Mock Control	64.13 ± 2.87^bA^	180.25 ± 6.57^aA^	60.05 ± 3.52^aA^	83.28 ± 0.75^aA^	36.86 ± 1.77^aA^	41.61 ± 1.49^aA^
Mock BLP	144.95 ± 1.29^aA^	227.25 ± 7.42^bB^	62.05 ± 2.33^aA^	95.87 ± 3.74^bAB^	50.96 ± 1.42^bB^	56.66 ± 1.98^bB^
Infection Control	219.06 ± 6.29^cB^	261.95 ± 5.04^cC^	86.47 ± 2.28^bB^	102.12 ± 4.05^bBC^	50.40 ± 2.29^bB^	55.22 ± 1.60^bB^
Infection BLP	210.34 ± 8.89^cB^	269.41 ± 4.46^cC^	86.33 ± 2.34^bB^	113.66 ± 4.94^cC^	52.71 ± 1.02^bB^	59.77 ± 1.74^bB^

Note: The different lowercases and capitals indicated significant difference at level of *P* < 0.05 and *P* < 0.01

### Effect of BLP on the tissue ALV titer of infected chickens

**Fig. 3:**
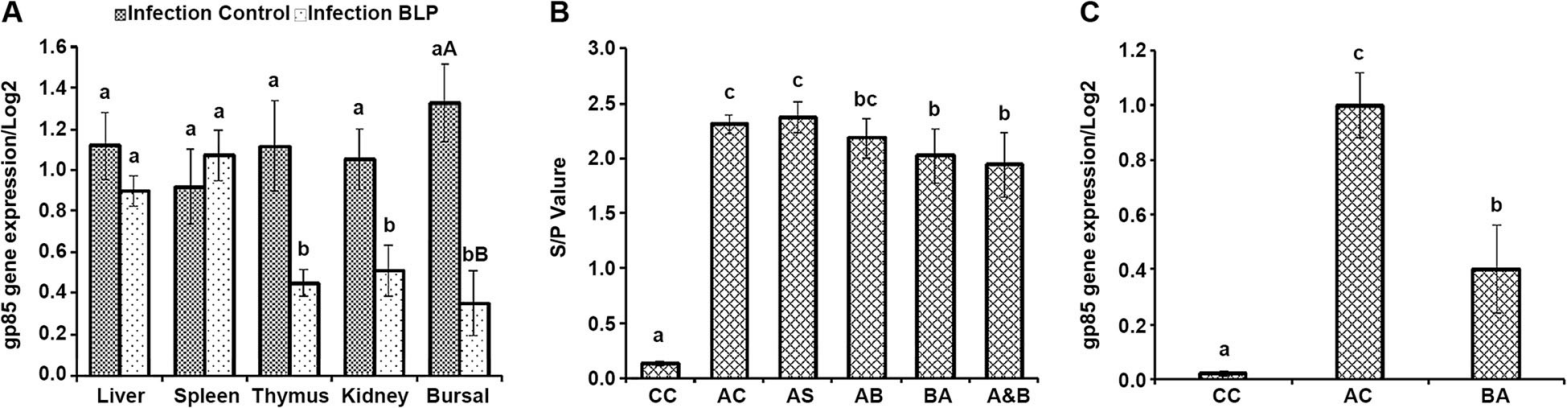
The antiviral action of BLP in vivo and in vitro. A Effect of BLP on ALV *gp85* gene relative expression measured by qRT-PCR in different tissues in vivo at 42 d of age (Log2). **B** Influence of BLP on the expression of p27 antigen at different stages of virus infection in vitro (mean ± SE). The groups in the figure were cell control (CC), ALV control (AC), ALV infection 2 h before BL21 supernatant incubation (AS), ALV infection 2 h before BLP incubation (AB), BLP incubation 2 h before ALV infection (BA), ALV and BLP together (A&B). The final concentrations of BLP were all 50 μg/mL in these three administrations (AB, BA, A&B). **C** ALV *gp85* gene relative expression in different groups measured with qRT-PCR in vitro (Log2). DF-1 cells with or without ALV inoculation were used as ALV control (AC) and cell control (CC), respectively. The different lowercases and capitals indicated significant difference at level of *P* < 0.05 and *P* < 0.01. Data were expressed from three independent experiments

The expression level of the ALV *gp85* gene in different tissues in ALV-infected chickens was assayed by qRT-PCR at 4 2d of age (Fig. 3a). Unexpectedly, weekly injection with purified BLP resulted in a significant reduction of ALV gp85 gene expression in the thymus (*P* < 0.05), kidney (*P* < 0.05) and BF (*P* < 0.01), showing the favorable antiviral action in vivo of this novel BLP.

### BLP antiviral action in vitro

In order to explore the antiviral action of BLP in vitro, BLP was assayed under different conditions. All treatments were applied to cells in vitro and antiviral activity was detected by ELISA for ALV p27 antigen. The results showed that the p27 *S*/*P* value of the BA group was significantly lower than the virus control, and also lower than that of AB and A&B groups (Fig. 3b). This means that BLP might inhibit virus adsorption onto DF-1 cells. The expression of p27 in the AB group was lower than the virus control but the difference was not significant (*P* > 0.05), which indicated that treatment with BLP after virus inoculation did not significantly decrease virus infection.

The expression of the ALV *gp85* gene in cells with different treatments was detected by qRT-PCR (Fig. 3c). The expression of the ALV *gp85* gene decreased significantly after treatment with 50 μg/mL BLP (*P* > 0.05).

## Discussion

A multitude of important viruses including ALVs [14], IBDV, avian reovirus, Marek’s disease virus (MDV), hemorrhagic enteritis virus [15] and avian reticuloen-dotheliosis virus (REV) [4] have been implicated in avian immunosuppression. Until now, there are limited reports of the in vivo or in vitro action of naturally-occurring substances and heterosynthetic immunomodulators in chickens infected with viruses causing immune-suppression. A previous report revealed that dietary allicin supplementation ameliorated REV-induced dysplasia and immune dysfunction in infected chickens [16]. Taishan *Pinus massoniana* pollen polysaccharide and propolis generated positive effects on improving the immune effectiveness of immune-suppressed chickens caused by co-infection of REV and ALV-J [17]. Treatment with synthetic thymulin by daily subcutaneous injection failed to modify the time course of Marek’s disease (MD) and did not prevent the development of macroscopic tumors [18]. Low MW poly-saccharides from *Grateloupia filicina* [19], lithium chloride [20] and baicalin [21] showed antiviral activity against ALV-J in vitro. Previous studies have been conducted on the positive effects of BS and BLP on immunity in different animals [7, 11, 12, 22], indicating the remarkable potential of BS and its analogs. However, to our knowledge, the effect of BS and BLP on poultry flocks infected with pathogens resulting in immune suppression has not been reported.

Interestingly, we found that BLP injection led to body weight regain in ALV-infected chickens. It is worth noting that BLP given to uninfected chickens showed no significant increase in growth performance. The effect of BLP injection on growth performance and its regulatory mechanism requires further research.

Until now, there have not been any reports on the effect of BLPs on animal humoral immune responses against active vaccines. In a previous report, we found that the vertical infection of the recombinant ALV strain FJ15HT0 in chickens exerted a negative effect on the humoral immune response to active vaccines but not to inactive vaccines [3]. Coincidentally, in this report we found that injection of our novel BLP maintained the antibody titer peak against the active NDV vaccine in ALV FJ15HT0 infected chickens but not mock birds (Fig. 2c), revealing the immune modulating effect of this novel BLP to withstand the immune suppression induced by the ALV FJ15HT0 strain. This result may be explained by the suppression of BLP on ALV replication in immune organs (Fig. 3a). In keeping with previous reports [11–13], we found that the injection of BLP led to a significant boost in the antibody titer against AIV-H_5_ inactivated vaccine inoculated subcutaneously in mock SPF chickens but not in birds infected with ALV (Fig. 2d). This result implied that differences in the mechanism of action of BLP might exist between different vaccine types.

In the present study, increased production of IL-2 and IFN-γ in ALV-infected chickens reflected an interaction between the ALV FJ15HT0 strain and host, and the result aligns with previous studies [23]. The increased production of IL-2 and IFN-γ (Th1 cytokines) suggested an induction of the Th1 axis in response to BLP [13]. These findings demonstrated that BLP may have immune-regulatory functions via cytokine promotion.

As a member of the retroviruses, ALV shares a similar replication mechanism with human immunodeficiency virus (HIV) [24]. Antiretroviral therapy (ART) has significantly modified the history of HIV infection and is responsible for delaying virus replication and preserving the CD_4_^+^ T cell count in humans [25, 26]. Unexpectedly, we found that BLP was an inhibitor of ALV, especially in the central immune organs, the thymus and BF, in vivo. This novel BLP also showed significant antiviral action in vitro, especially in the pre-infection phase. This result is interesting and to our knowledge has not been previously reported. The action of BLP is not species-specific [9, 11, 22], and the mechanism of action of BLP as an ALV inhibitor may have relevance for HIV control.

## Conclusions

Overall, the comprehensive action of this novel BLP in ALV-infected chickens was remarkably positive, including significant growth-promoting action, an immunity-regulating effect and antiviral activity, without significant untoward effects, displaying remarkable potential for future utilization. The effect of this BLP on other retroviruses or viruses associated with immunosuppression is worthy of further research.

## Methods

### Virus background

The recently isolated recombinant ALV FJ15HT0 [3], which associated with immunity and growth suppression in infected chickens, was used in this study.

### Construction of recombinant expression plasmid and expression in *E. coli*

A fusion gene BLP was generated by concatenating the oligonucleotides encoding a duplicated Lys-Asn-Pro-Tyr peptide, a linker (GGGG), and albumin-binding peptide [27]. The gene was amplified by polymerase chain reaction (PCR) using primers obtained from Sangon Co. Ltd. (Shanghai, China). The PCR conditions were 30 cycles of 30 s at 95 °C, 30 s at 55 °C, and 1 min at 72 °C. The amplified gene was cloned into the prokaryotic expression vector pET-32a(+) (Sangon, Shanghai, China) and the resulting BLP expression plasmids were transformed into *E. coli* strain BL21 (DE3) (Stratagene, La Jolla, CA, USA). After induction with 24 μg/mL isopropyl β-D-thiogalactopyrano-side (IPTG; Sigma, St. Louis, MO, USA) and 0.01 g/mL saccharose (Sigma, St. Louis, MO, USA) for 20 h at 24.5 °C, the BLP fusion conjugate was purified using Ni-NTA affinity resin (ThermoFisher, Shanghai, China). The recombinant bacterial protein was separated on a 12% (v/v) SDS-PAGE gel and a 28 kDa band corresponding to the recombinant hybrid polypeptide, including the vector fusion protein (20 kDa) and the hybrid polypeptide (approx. 8 kDa), was isolated. Protein concentration was determined with a bicinchoninic acid (BCA) assay.

### Animal experimental design

The treatments applied to birds in different groups are shown in Table 2. In total sixty one-day-old specific-pathogen-free (SPF) chicken eggs (SPAFAS poultry company, Jinan, China) were randomly divided into four groups (15 eggs per group). Birds in different groups were hatched separately and then raised separately in isolation feeding devices for 42 days (d). The ALV isolate or placebo (DMEM) were inoculated via the vitellicle at day 6 of embryonation in different groups. Infection of inoculated SPF chickens was confirmed by the shedding of ALV on the first day after hatching. At day 1 and day 14 of age, all experimental animals were simultaneously inoculated with attenuated Newcastle disease virus (NDV) vaccine (Harbin Veterinary Research Institute, a plume, 0.2 mL, Strain LaSota, via eye and nasal drops) and inactivated AIV H_5_ subtype vaccine (Harbin Veterinary Research Institute, a plume, 0.3 mL, Strains Re-6 and Re-7, subcutaneously). The BLP/placebo was subcutaneously injected weekly.

**Table 2:** Treatment of chickens in different groups

Group	ALV/placebo Inoculation	Vaccination	BLP/placebo Inoculation
Mock Control	DMEM	NDV + AIV	purified BL21 culture
Mock BLP	DMEM	NDV + AIV	50 μg purified BLP
Infection Control	2.0 × 10^5^ TCID_50_	NDV + AIV	purified BL21 culture
Infection BLP	2.0 × 10^5^ TCID_50_	NDV + AIV	50 μg purified BLP

The animals were maintained according to the National Standards for Laboratory Animals of China (GB 14925–2010). All animal tests were approved by the Institutional Animal Care and Use Committee and were conducted following the guidelines of the Institutional Biosafety Committee at Fujian Agricultural and Forestry University.

### Measurement of body weight and organ indices

To compare the effects of our novel BLP on growth retardation and immunosuppression induced by ALV infection, individual body weights (BWs) were measured at days 1, 14, 21, 28, 35 and 42 in the four experimental groups. At 42 d of age, animals were sacrificed by a lethal intravenous injection of beuthanasia (0.3 mL/kg) after anesthesia with ketamine- Xylazine. The samples of liver, kidney, thymus, spleen and BF were excised from all chicks and weighed to establish organ indices, which were calculated as organ weight (wet weight, mg)/BW (g) × 100%.

### Detection of specific antibodies and cytokines in chicken serum

Serum samples were collected from each bird at 1, 14, 28 and 42 days of age. Serum antibody titers against NDV and AIV were assayed by hemagglutination inhibition (HI) assay according to the OIE manual [28, 29]. The presence of the cytokines IL-4 and interferon-γ (IFN-γ) in serum samples from vaccinated birds at 14 d and 28 d was examined using commercially available chicken cytokine ELISA kits (Jingmei Corporation, China). In each assay, control recombinant chicken cytokine was diluted over the recommended detection range to generate a standard curve. Sample concentrations were interpolated from the standard curve.

### In vivo anti-viral efficacy testing

RNA was extracted from tissues of all experimental animals at 42 d of age and quantitative real-time PCR (qRT-PCR) for selected genes was performed as reported previously [30]. The qRT-PCRs were performed using SYBR Premix ExTaq™ II (Perfect Real Time) (TAKARA, Japan) following the manufacturer’s instructions. Each reaction contained SYBR Green I qRT-PCR primer sets specific for the *gp85* gene of the FJ15HT0 strain. Chicken β-actin and glyceraldehyde-3-phosphate dehydrogenase (G3PDH) genes served as internal controls. We performed qRT-PCR on an ABI7900HT and each sample was run in triplicate. Gene expression levels were calculated using the 2^−ΔΔCt^ method.

### In vitro anti-viral efficacy testing

To reveal the antiviral action of this novel BLP in vitro, a cell-based experiment was designed as shown in Table 3. Chicken embryo fibroblast cell lines, DF1 cells (American Type Culture Collection, USA) (4 × 10^5^ cells/well) grown in 6 well plates were randomly allocated to different treatment groups with 12 samples per group. One day after the cells were seeded at a ratio of 80:1, the cells in all groups except the CC group were incubated with ALV FJ15HT0 (5 × 10^4^ TCID_50_) for 45 min. Purified BLP (50 μg) was injected before ALV inoculation (BA), together with ALV (A&B) or after ALV infection (AB) to investigate the action phase of BLP. All assays included medium-only control (CC), ALV infection only control (AC) and purified supernatant of BL21 with null vector control (AS) in quadruplicate. Infected cells were incubated in the presence of BLP (AB, BA, A&B groups) or placebo (AS group) in culture medium until 2 days post infection (dpi). The medium containing BLP or placebo was then replaced with normal culture medium and cells were incubated until 5 dpi. To evaluate the ALV titer, supernatants and cells were collected at 5 dpi from quadruplicate wells. The titer of ALV-specific antigen p27 in all experimental groups of DF1 cells was examined using a commercially available ALV antigen test kit (IDEXX Corporation, America), following the manufacturer’s instructions. The *gp85* gene expression levels in cells in groups CC, AC and BA were measured by qRT-PCR as described above.

**Table 3:** Treatment of cells in different groups

Group	ALV/placebo Dosage	BLP/placebo Dosage	Action Phase
Cell Control (CC)	DMEM	N	N
ALV Control (AC)	5 × 10^4^ TCID_50_	N	N
ALV + BL21 supernatant (AS)	5 × 10^4^ TCID_50_	purified BL21 culture	2 h after ALV infection
ALV + BLP (AB)	5 × 10^4^ TCID_50_	50 μg purified BLP	2 h after ALV infection
BLP + ALV (BA)	5 × 10^4^ TCID_50_	50 μg purified BLP	2 h before ALV infection
ALV and BLP together (A&B)	5 × 10^4^ TCID_50_	50 μg purified BLP	Together with ALV infection

### Statistical analysis

The data are presented as means ± standard error (SE) of the mean. Statistical analyses were performed by one-way analysis of variance (ANOVA) at a significance level of 0.05 and 0.01 using the SPSS statistical software package (version 19.0; SPSS Company, Chicago, IL, USA).

## Data Availability

All data included in this study is available upon request to the corresponding author.

## Abbreviations

AIV: Avian influenza virus
ALVs: Avian leukosis viruses
ART: Antiretroviral therapy
BCA: Bicinchoninic acid
BF: Bursa of Fabricius
BLP: Bursin-like fusion peptide
HI: Hemagglutination inhibition
HIV: Human immunodeficiency virus
IBDV: Infectious bursal disease virus
JEV: Japanese encephalitis virus
MDV: Marek’s disease virus
NDV: Newcastle disease virus
QRT-PCR: Quantitative reverse transcription-polymerase chain reactions
REV: Reticuloendotheliosis virus
SE: Standard error
SPF: Specific-pathogen-free

## References

[1] Fadly AM: **Leukosis/sarcoma group**. In: *Diseases of Poultry.* edn. Edited by Saif YM. Ames, Iowa; 2003: 465–516.

[2] Landman WJ, Post J, Boonstra-Blom AG, Buyse J, Elbers AR, Koch G (2002). Effect of an in ovo infection with a Dutch avian leukosis virus subgroup J isolate on the growth and immunological performance of SPF broiler chickens. Avian Pathol.

[3] Wu X, Zhao J, Zeng Y, Wu Y, Wang Q, Wu B, Huang Y (2017). A novel avian retrovirus associated with lymphocytoma isolated from a local Chinese flock induced significantly reduced growth and immune suppression in SPF chickens. Vet Microbiol.

[4] Witter RL, Fadly AM (2001). Reduction of horizontal transmission of avian leukosis virus subgroup J in broiler breeder chickens hatched and reared in small groups. Avian Pathol.

[5] Jing W, Zhou J, Wang C, Qiu J, Guo H, Li H (2018). Preparation of the secretory recombinant ALV-J gp85 protein using Pichia pastoris and its Immunoprotection as vaccine antigen combining with CpG-ODN adjuvant. Viral Immunol.

[6] Payne LN, Nair V (2012). The long view: 40 years of avian leukosis research. Avian Pathol.

[7] Audhya T, Kroon D, Heavner G, Viamontes G, Goldstein G (1986). Tripeptide structure of bursin, a selective B-cell-differentiating hormone of the bursa of fabricius. Science.

[8] Brand A, Gilmour DG, Goldstein G (1976). Lymphocyte-differentiating hormone of bursa of fabricius. Science.

[9] Baba T, Kita M (1977). Effect of extracts of the bursa of Fabricius on IgG antibody production in hormonally bursectomized chickens. Immunology.

[10] Gagnon L, DiMarco M, Kirby R, Zacharie B, Penney CL (2000). D-LysAsnProTyr tetrapeptide: a novel B-cell stimulant and stabilized bursin mimetic. Vaccine.

[11] Wang C, Wen WY, Su CX, Ge FF, Dang ZG, Duan XG, Cao RB, Zhou B, Chen PY (2008). Bursin as an adjuvant is a potent enhancer of immune response in mice immunized with the JEV subunit vaccine. Vet Immunol Immunopathol.

[12] Wang C, Li X, Wu T, Li D, Niu M, Wang Y, Zhang C, Cheng X, Chen P (2014). Bursin-like peptide (BLP) enhances H9N2 influenza vaccine induced humoral and cell mediated immune responses. Cell Immunol.

[13] Cai MH, Zhu F, Wu HC, Shen PP (2014). A new recombinant hybrid polypeptide and its immunologic adjuvant activity for inactivated infectious bursal disease vaccine. Biotechnol Lett.

[14] Ignjatovic J, Fraser RA, Bagust TJ (1986). Effect of lymphoid leukosis virus on performance of layer hens and the identification of infected chickens by tests on meconia. Avian Pathol.

[15] Sharma JM, Karaca K, Pertile T (1994). Virus-induced immunosuppression in chickens. Poult Sci.

[16] Wang L, Jiao H, Zhao J, Wang X, Sun S, Lin H (2017). Allicin alleviates Reticuloendotheliosis virus-induced immunosuppression via ERK/mitogen-activated protein kinase pathway in specific pathogen-free chickens. Front Immunol.

[17] Li B, Wei K, Yang S, Yang Y, Zhang Y, Zhu F, Wang D, Zhu R (2015). Immunomodulatory effects of Taishan Pinus massoniana pollen polysaccharide and propolis on immunosuppressed chickens. Microb Pathog.

[18] Quere P, Dambrine G, Bach MA (1989). Influence of thymic hormone on cell-mediated and humoral immune responses in Marek's disease. Vet Microbiol.

[19] Sun Yuhao, Chen Xiaolin, Cheng Ziqiang, Liu Song, Yu Huahua, Wang Xueqin, Li Pengcheng (2017). Degradation of Polysaccharides from Grateloupia filicina and Their Antiviral Activity to Avian Leucosis Virus Subgroup J. Marine Drugs.

[20] Qian K, Cheng X, Zhang D, Shao H, Yao Y, Nair V, Qin A (2018). Antiviral effect of lithium chloride on replication of avian leukosis virus subgroup J in cell culture. Arch Virol.

[21] Qian K, Kong ZR, Zhang J, Cheng XW, Wu ZY, Gu CX, Shao HX, Qin AJ (2018). Baicalin is an inhibitor of subgroup J avian leukosis virus infection. Virus Res.

[22] Abiko T, Sekino H (1994). Syntheses and effect of bursin and it analogs on the reduced B lymphocytes of uremic patients. Biotechnol Ther.

[23] Gao Y, Liu Y, Guan X, Li X, Yun B, Qi X, Wang Y, Gao H, Cui H, Liu C (2015). Differential expression of immune-related cytokine genes in response to J group avian leukosis virus infection in vivo. Mol Immunol.

[24] Swanstrom R, Graham WD, Zhou S (2017). Sequencing the biology of entry: the retroviral env gene. Curr Top Microbiol Immunol.

[25] Malta M, da Silva CM, Magnanini MM, Wirtz AL, Perisse AR, Beyrer C, Strathdee SA, Bastos FI (2015). Improvement of HAART in Brazil, 1998-2008: a nationwide assessment of survival times after AIDS diagnosis among men who have sex with men. BMC Public Health.

[26] Venturini A, Giannini B, Montefiori M, Di Biagio A, Mazzarello G, Cenderello G, Giacomini M, Merlano C, Orcamo P, Setti M (2014). Quality of life of people living with HIV, preliminary results from IANUA (investigation on antiretroviral therapy) study. J Int AIDS Soc.

[27] Kim D, Jeon H, Ahn S, Choi WI, Kim S, Jon S (2017). An approach for half-life extension and activity preservation of an anti-diabetic peptide drug based on genetic fusion with an albumin-binding aptide. J Control Release.

[28] Epizooties OId: Newcastle disease. Manual of Standards for Diagnostic Tests and Vaccines for Terrestrial Animals. In*.* Paris, France; 2008: 465–481.

[29] Epizooties OId: Avian influenza. Manual of Standards for Diagnostic Tests and Vaccines for Terrestrial Animals. In*.* Paris, France; 2008: 465–481.

[30] Rawat P, Mitra D (2011). Cellular heat shock factor 1 positively regulates human immunodeficiency virus-1 gene expression and replication by two distinct pathways. Nucleic Acids Res.

